# The Effectiveness of Methylene Blue in Adult Shock: A Systematic Review, Meta-Analysis, and Trial Sequential Analysis of Randomized Controlled Trials

**DOI:** 10.3390/jcm15124481

**Published:** 2026-06-10

**Authors:** David Rene Rodríguez-Lima, Adelaida Rodríguez-Villegas, Juan Diego Toro Egas, Esther María Campo Bautista

**Affiliations:** 1 Critical and Intensive Care Medicine, Hospital Universitario Mayor-Méderi, Bogotá 111411, Colombia; 2 Clinical Research Group, School of Medicine and Health Sciences, Universidad del Rosario, Bogotá 111411, Colombia; 3 School of Medicine and Health Sciences, Universidad del Rosario, Bogotá 111411, Colombia; adelaida.rodriguezv@urosario.edu.co (A.R.-V.); juan.toro@urosario.edu.co (J.D.T.E.); 4 Cardiology Department, La Cardio, Bogotá 111411, Colombia; emcampo@lacardio.org

**Keywords:** methylene blue, shock, septic shock, vasoplegia, vasopressors, randomized controlled trial, systematic review, meta-analysis, trial sequential analysis

## Abstract

**Background/Objectives:** Methylene blue (MB) has re-emerged as an adjunctive vasopressor-sparing therapy in vasoplegic shock states, with emerging evidence supporting its hemodynamic benefits; however, its effect on mortality remains uncertain. We systematically evaluated the effectiveness of MB versus standard therapy in adults with circulatory shock. **Methods:** We performed a systematic review and meta-analysis of randomized controlled trials registered in PROSPERO (CRD420261326534) and reported according to PRISMA. MEDLINE, Embase, and the Cochrane Library were searched through February 2026. An additional AI-assisted supplementary search was conducted to minimize the risk of missing eligible studies. Eligible studies enrolled adults with shock and compared MB with standard therapy or placebo. The primary outcome was 28–30-day all-cause mortality. Secondary outcomes were renal replacement therapy (RRT), hospital length of stay, and intensive care unit (ICU) length of stay. Risk of bias was assessed with RoB 2. **Results:** Nine randomized trials involving 535 participants met the eligibility criteria; most evaluated septic shock, while one trial included post-cardiac surgery vasoplegic shock. Eight trials contributed to the quantitative synthesis of mortality. MB was not associated with a statistically significant reduction in short-term mortality. Secondary analyses also did not demonstrate significant pooled effects for RRT, hospital length of stay, or ICU length of stay, although several individual trials reported faster hemodynamic improvement and reduced vasopressor exposure with MB. Overall confidence in the pooled estimates was limited by small sample sizes, clinical heterogeneity, imprecision, and risk-of-bias concerns in some studies. **Conclusions:** Current randomized evidence does not demonstrate a clear mortality or resource use benefit of MB in adult shock, despite signals of hemodynamic improvement. MB appears promising as an adjunctive therapy, but adequately powered, methodologically rigorous trials are required before its routine early use can be recommended.

## 1. Introduction

Shock is a state of circulatory failure that results in inadequate tissue perfusion, cellular hypoxia, and progressive organ dysfunction, and it remains associated with substantial morbidity and mortality in critically ill adults [1,2]. Approximately one-third of patients admitted to intensive care units are affected by some form of shock, although the epidemiology and prognosis vary according to the underlying mechanism [3,4,5,6].

Four broad pathophysiological categories are commonly recognized: hypovolemic, cardiogenic, obstructive, and distributive shock [1]. In clinical practice, these mechanisms may overlap, and diagnosis relies on a combination of clinical examination, laboratory testing, hemodynamic assessment, and serial evaluation of organ dysfunction [6,7]. Management focuses on rapid identification of reversible causes, individualized resuscitation, and timely use of vasoactive support when hypotension persists despite initial measures [1,8,9].

In refractory vasoplegia, dysregulated nitric oxide signaling plays a central role. Excess nitric oxide activates soluble guanylate cyclase, increases cyclic guanosine monophosphate (cGMP), and promotes pathological vasodilation in vascular smooth muscle cells. Methylene blue inhibits the nitric oxide–soluble guanylate cyclase–cGMP pathway and may therefore restore vascular tone and reduce vasopressor requirements [10,11,12].

Despite this biological rationale, methylene blue (MB) is still generally considered a rescue or adjunctive therapy rather than part of first-line management [9,10]. Existing evidence has mainly focused on septic shock, and previous syntheses have either excluded newer randomized trials or combined randomized and non-randomized evidence [11,13,14]. In addition, the applicability of MB across different vasoplegic states, including post-cardiac surgery vasoplegia, remains uncertain.

The aim of this systematic review and meta-analysis was to evaluate the efficacy of MB versus standard therapy on clinically relevant outcomes in adult patients with circulatory shock.

## 2. Materials and Methods

### 2.1. Study Protocol

The review protocol was registered in the International Prospective Register of Systematic Reviews (PROSPERO) under registration number CRD420261326534. The review was conducted and reported in accordance with the Preferred Reporting Items for Systematic Reviews and Meta-Analyses (PRISMA) statement.

### 2.2. Search Strategy and Eligibility Criteria

We searched MEDLINE, Embase, and the Cochrane Library (including CENTRAL) from inception through February 2026. Search terms combined controlled vocabulary (MeSH headings in MEDLINE; Emtree terms in Embase) and free-text terms related to “methylene blue,” “methylthioninium chloride,” “shock,” “septic shock,” “vasoplegic shock,” “vasoplegia,” and related terms. No language restrictions or date limits were applied. Reference lists of all included studies and relevant systematic reviews were screened manually to identify additional eligible records. The full, database-specific search strategies are provided in Supplementary Table S1.

Eligible studies were randomized controlled trials enrolling adult patients (≥18 years) with any form of circulatory shock and comparing MB with standard care, placebo, or another vasoactive strategy. We excluded studies evaluating prophylactic perioperative MB administration, non-randomized designs, conference abstracts without full text, and duplicate reports.

### 2.3. Study Selection and Data Extraction

Two reviewers independently screened titles and abstracts, assessed full texts for eligibility, and extracted data using a standardized form. As a supplementary retrieval strategy, Claude Sonnet 4.6 (Anthropic, claude.ai), a large language model-based AI assistant, was queried using structured clinical prompts related to methylene blue and circulatory shock to identify potentially eligible records that may not appear in conventional keyword-based database searches. Claude was used exclusively as a supplementary retrieval aid; it was not used for study selection, data extraction, risk-of-bias assessment, or any analytical task. The AI-assisted search retrieved 14 additional records. To ensure methodological transparency and minimize the risk of bias, all AI-retrieved records underwent the same dual-reviewer eligibility assessment applied to database-retrieved records. A random sample of 50% of AI-retrieved records was independently cross-checked against primary database results to identify potential duplicates and verify retrieval accuracy. No AI-retrieved record was accepted without full-text human review and formal eligibility adjudication. Disagreements at any stage were resolved by discussion and, when necessary, by adjudication by a third reviewer.

Extracted variables included study design, country, population, shock phenotype, sample size, intervention and comparator details, follow-up duration, and outcomes of interest.

### 2.4. Outcomes

The primary outcome was all-cause mortality at 28–30 days. Secondary outcomes included the need for renal replacement therapy (RRT), hospital length of stay, and ICU length of stay.

### 2.5. Risk-of-Bias Assessment

Risk of bias was evaluated with the Cochrane RoB 2 tool for randomized trials [15]. The following domains were assessed: bias arising from the randomization process, bias due to deviations from intended interventions, bias due to missing outcome data, bias in measurement of the outcome, and bias in selection of the reported result. Each study was judged as low risk, some concerns, or high risk of bias.

### 2.6. Statistical Analysis

Study characteristics were summarized descriptively. For dichotomous outcomes, pooled effects were expressed as odds ratios (ORs) with 95% confidence intervals (CIs). For continuous outcomes, standardized mean differences (SMDs) with 95% CIs were used when needed to account for differences in scale or variance reporting across studies.

Heterogeneity was evaluated using Cochran’s Q and the I^2^ statistic. A random-effects model was favored when clinical or statistical heterogeneity was present; otherwise, fixed-effect methods were considered. Leave-one-out sensitivity analyses were performed for the primary outcome. Publication bias was explored visually with a funnel plot, recognizing the limited interpretability of small meta-analyses. All analyses were performed in RStudio (version 4.3.1).

### 2.7. Trial Sequential Analysis

Trial sequential analysis (TSA) was performed for the primary outcome using the R TSA package to evaluate the robustness of cumulative evidence and the risk of random error. Analyses used a two-sided alpha of 0.05 and 80% power.

## 3. Results

### 3.1. Study Selection and Characteristics

**Figure 1:**
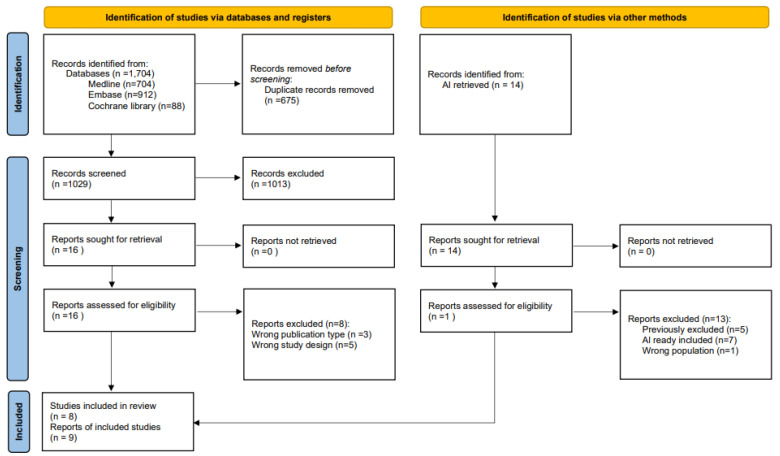
PRISMA 2020 flow diagram for study selection.

The database search identified 1704 records, and an additional 14 records were retrieved through an AI-assisted supplementary search. After duplicate removal and screening, nine randomized trials met the inclusion criteria for the qualitative synthesis [16,17,18,19,20,21,22,23,24]. One head-to-head trial comparing MB with vasopressin did not contribute data to the pooled outcomes of interest, leaving eight trials for the quantitative synthesis of mortality. The review process is summarized in Figure 1.

The included trials enrolled 535 participants overall and were published between 2001 and 2025. Most studies evaluated septic shock, whereas one trial assessed post-cardiac surgery vasoplegic shock. MB regimens varied considerably across studies, ranging from intravenous bolus-only strategies to prolonged infusions over several hours or days. Standard therapy also differed across trials and included varying combinations of fluids, norepinephrine, vasopressin, hydrocortisone, and protocolized hemodynamic monitoring. The general characteristics of the included studies are summarized in Table 1.

### 3.2. Risk of Bias

**Figure 2:**
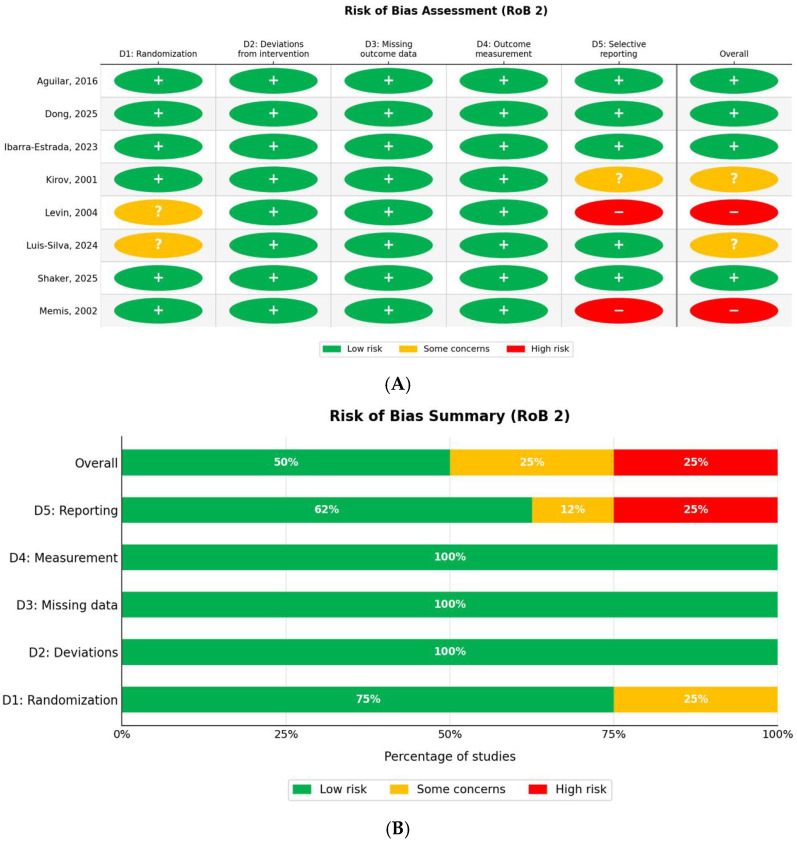
(**A**) Risk-of-bias assessment using the Cochrane RoB 2 tool for individual trials. (**B**) Summary distribution of RoB 2 judgments across domains.

RoB 2 assessment showed overall moderate methodological limitations. Four studies were judged to be at low risk of bias, two raised some concerns, and two were considered at high risk of bias for the primary outcome. Concerns were mainly related to the randomization process and selective reporting, whereas bias due to deviations from intended interventions, missing outcome data, and outcome measurement was generally low across studies (Figure 2A,B).

### 3.3. Primary Outcome: 28–30-Day All-Cause Mortality

**Figure 3:**
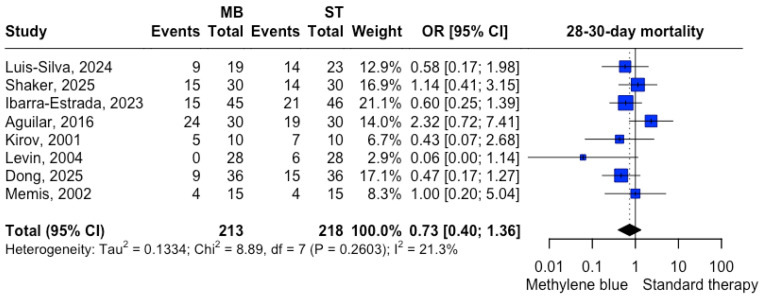
Forest plot for short-term mortality in septic shock trials. Squares represent individual study estimates (area proportional to study weight); diamond represents the pooled effect estimate; dashed vertical line indicates no effect (OR = 1).

In the pooled analysis, MB was not associated with a statistically significant reduction in short-term mortality. The overall direction of effect favored MB, but the confidence interval crossed the null value, indicating substantial uncertainty around the true treatment effect. Heterogeneity for the primary analysis was low (Figure 3).

**Figure 4:**
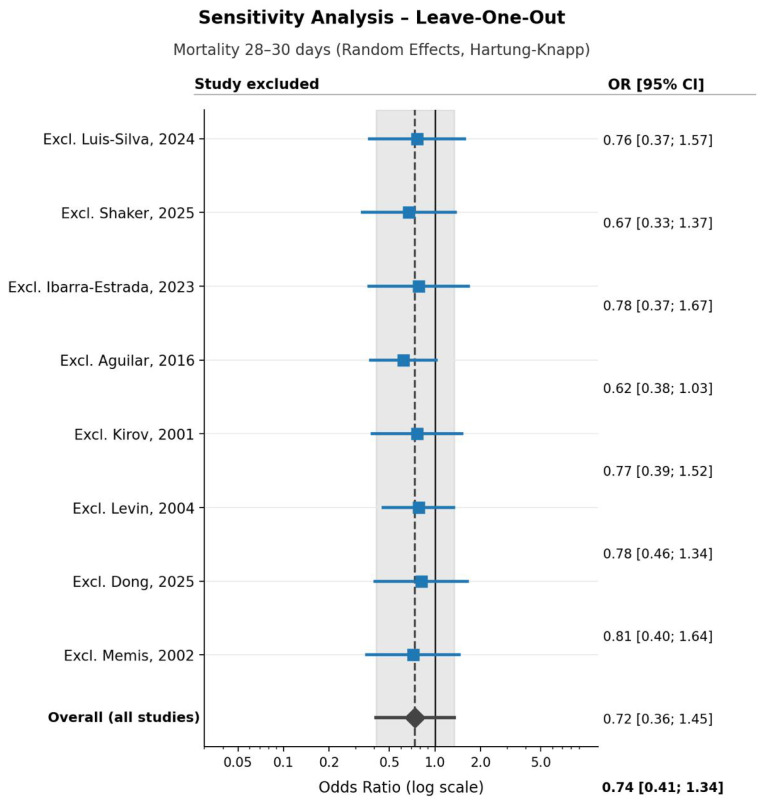
Leave-one-out sensitivity analysis for 28–30-day mortality. Squares represent individual study estimates; diamond represents the pooled effect estimate; dashed line indicates no effect.

Leave-one-out analysis showed that exclusion of any single study did not materially change the overall conclusion, supporting the stability of the non-significant result. Similarly, subgroup analyses restricted to septic shock were directionally consistent with the main analysis and did not identify a clear mortality benefit (Figure 4).

### 3.4. Secondary Outcomes

**Figure 5:**
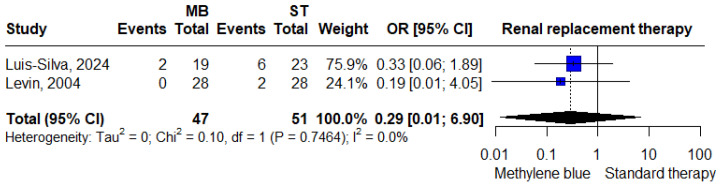
Forest plot for renal replacement therapy. Squares represent individual study estimates; diamond represents the pooled effect estimate; dashed line indicates no effect.

**Figure 6:**
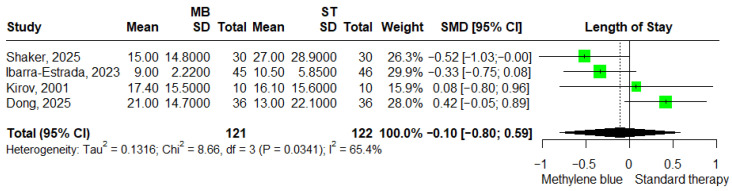
Forest plot for hospital length of stay. Squares represent individual study estimates; diamond represents the pooled effect estimate; dashed line indicates no effect.

**Figure 7:**
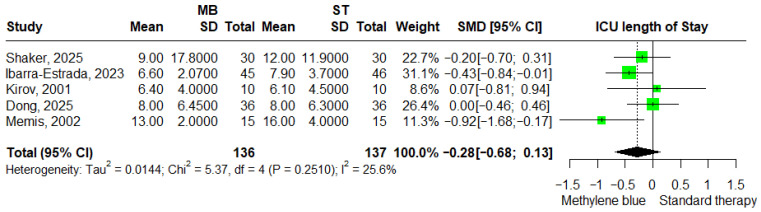
Forest plot for ICU length of stay. Squares represent individual study estimates; diamond represents the pooled effect estimate; dashed line indicates no effect. Squares represent individual study estimates; diamond represents the pooled effect estimate; dashed line indicates no effect.

MB was not associated with a significant reduction in the need for RRT in the pooled analysis, Figure 5. Likewise, pooled analyses for hospital and ICU length of stay did not show statistically significant differences between groups, Figure 6 and Figure 7, although individual trials suggested shorter stays in some septic shock populations receiving early adjunctive MB.

### 3.5. Publication Bias and Trial Sequential Analysis

**Figure 8:**
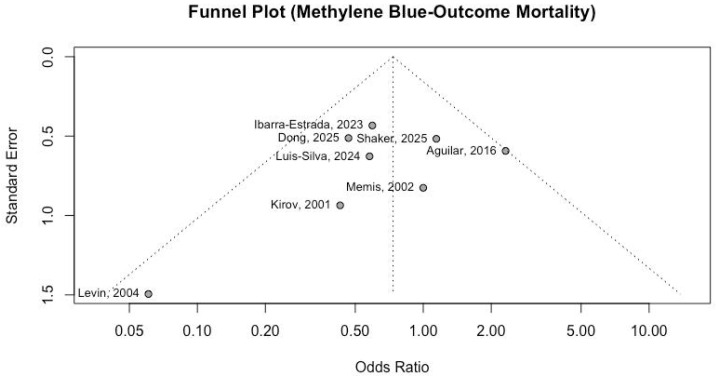
Funnel plot for 28–30-day mortality. Dashed lines represent the trial sequential analysis (TSA) monitoring boundaries for benefit and futility.

The funnel plot for mortality was difficult to interpret because of the small number of studies and the clinical diversity of the included trials; no strong conclusion regarding publication bias can therefore be made (Figure 8). However, when the analysis was restricted to the eight septic shock trials, excluding Levin et al. (2004) [22], which evaluated vasoplegic post-cardiac surgery shock, the funnel plot showed no evidence of publication bias.

**Figure 9:**
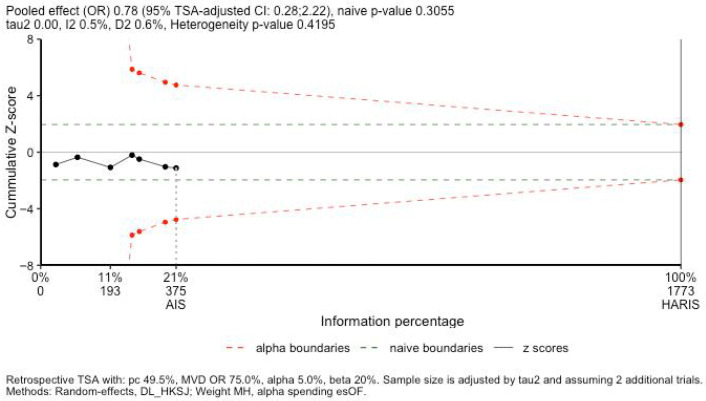
Trial sequential analysis for mortality. Circle dots (•) represent individual study estimates in the funnel plot.

TSA was performed on the eight septic shock trials included in the primary mortality analysis (the Levin 2004 [22] post-cardiac surgery trial was excluded from TSA given its distinct population and its disproportionate graphical influence). The pooled OR for 28–30-day mortality was 0.73 (95% CI 0.40–1.36), indicating a directional trend favoring MB that did not reach statistical significance. Using an assumed relative risk reduction of 25% based on this observed OR, a two-sided alpha of 0.05, and 80% power, the heterogeneity-adjusted required information size (RIS) was estimated at 1773 patients. The cumulative information accrued across the eight trials represented a fraction of this threshold, and the Z-curve remained within the monitoring boundaries throughout, never crossing the conventional significance boundary, the trial sequential benefit boundary, or the futility boundary (Figure 9). These results confirm that the available evidence is substantially underpowered and that no definitive conclusion, in either direction, can currently be drawn regarding a mortality effect of MB.

### 3.6. Certainty of the Evidence

The overall confidence in the pooled evidence was limited. Small sample sizes, wide confidence intervals, heterogeneous dosing strategies, differences in comparators and co-interventions, and risk-of-bias concerns in several trials all reduced certainty. Even where point estimates favored MB, the precision of the available evidence was insufficient to support firm clinical recommendations (Table 2).

## 4. Discussion

This systematic review synthesized randomized evidence on MB for adult circulatory shock and found no statistically significant reduction in 28–30-day mortality. Similarly, pooled analyses did not demonstrate significant benefits for RRT use, hospital length of stay, or ICU length of stay. Nonetheless, multiple individual trials reported favorable hemodynamic effects, including improved arterial pressure, lower vasopressor exposure, and faster vasopressor discontinuation, suggesting that MB may exert physiologic benefits that have not yet translated into confirmed improvements in mortality or clinical outcomes. As detailed in Table 1, the most clinically compelling individual results include a significantly shorter time to vasopressor discontinuation with MB in Ibarra-Estrada et al. (2023) [19]—69 h versus 94 h in controls (*p* < 0.001)—and a mortality reduction from 21.4% to 0% observed in the vasoplegic post-cardiac surgery population of Levin et al. (2004) [22]. However, these findings stem from small, heterogeneous trials and should not be extrapolated to broader clinical practice without confirmation in adequately powered trials [13,19,23].

These findings are biologically plausible. By inhibiting the nitric oxide–soluble guanylate cyclase–cGMP pathway, MB may counteract pathological vasodilation and enhance vascular responsiveness to catecholamines [12]. This mechanism is especially relevant in vasoplegic states such as septic shock and post-cardiac surgery vasoplegia, where excessive nitric oxide-mediated vasodilation is a dominant pathophysiological feature [12,22]. However, hemodynamic improvement does not necessarily translate into survival benefit, particularly in multifactorial syndromes where outcome is also shaped by infection control, source management, organ support, myocardial dysfunction, and timing of intervention [8,13].

Our results are consistent with those of prior systematic reviews evaluating MB in septic shock. Ballarin et al. [11] identified a trend toward lower mortality with MB but noted that individual studies were markedly underpowered and that pooled estimates carried wide confidence intervals. More recently, Alkazemi et al. [14] conducted a meta-analysis of both randomized and prospective observational studies and similarly observed modest hemodynamic benefits without a definitive mortality signal. Fernando et al. [13] provided a narrative synthesis emphasizing that existing evidence supports MB as a rescue strategy in catecholamine-refractory vasoplegia, while cautioning against broader adoption in the absence of adequately powered trials. The present review extends these contributions by incorporating the most recent randomized evidence, including trials published through early 2026 [18,20,24], by restricting the quantitative synthesis to RCTs only, and by explicitly characterizing the information deficit through TSA.

The current evidence base has several important limitations. First, the literature is dominated by small, single-center trials, many of them explicitly designed as pilot studies with sample sizes insufficient for detecting clinically relevant mortality differences. Second, the included studies exhibited a high degree of clinical heterogeneity at two levels. At the population level, eight trials evaluated septic shock while one trial [22] enrolled patients with vasoplegic syndrome following cardiac surgery—two conditions that differ substantially in pathophysiology, baseline mortality risk, and treatment algorithms. Although subgroup analyses restricted to septic shock trials showed directionally consistent results, this population-level heterogeneity limits the internal validity of the overall pooled estimate and should be borne in mind when interpreting the results. At the intervention level, MB dosing regimens varied markedly across studies, including a 3 mg/kg continuous infusion over 6–48 h [16,23], a 4 mg/kg bolus with a 72-hour infusion [18], a single intravenous bolus of 1 mg/kg [20], and a 2 mg/kg bolus followed by infusions [21]. These differences in dose, administration route, and duration make it difficult to define an optimal MB regimen and may obscure or attenuate true treatment effects. Third, follow-up periods were heterogeneous across studies: several trials reported outcomes at 24, 72, or 96 h, whereas the pre-specified primary endpoint was 28–30-day all-cause mortality. This variability reduces the comparability of outcome data and complicates standardization in the quantitative synthesis. Fourth, some studies were open-label, and risk-of-bias concerns were not negligible, particularly regarding the randomization process and selective reporting. Fifth, the number of studies available for each secondary outcome was critically limited. Specifically, the pooled analysis of the need for renal replacement therapy (RRT) was based on only two studies—a sample far too small to support reliable meta-analytic inference; this finding should be interpreted with particular caution, and no clinical conclusion should be drawn from it in isolation. Similarly, the analyses for hospital and ICU length of stay were restricted to a small number of heterogeneous studies. Finally, and most critically, TSA demonstrated that the accrued information size remained far below the heterogeneity-adjusted required information size, and that the cumulative Z-curve did not cross conventional or trial sequential monitoring boundaries. This finding directly indicates that the available evidence is underpowered and inconclusive: the current data are insufficient to confirm or refute a mortality benefit, and the apparent absence of statistical significance should not be interpreted as proof of no effect.

This distinction between insufficient evidence and evidence of absence is fundamental to the correct interpretation of this review. TSA operates analogously to interim monitoring boundaries in adaptive clinical trials: when the cumulative Z-curve does not cross either the benefit or the futility boundary, and the accrued information remains below the required information size, no definitive conclusion can be drawn in either direction. In the present analysis, the observed OR of 0.78 (95% CI 0.28–2.22) reflects a consistent directional signal favoring MB across eight septic shock RCTs, yet the extremely wide confidence interval and the fact that only approximately 30% of the RIS of 1773 patients has been enrolled to date explain why statistical thresholds remain unmet. Crucially, the Z-curve did not cross the futility boundary either—meaning the data do not rule out a meaningful treatment effect. If the point estimate of OR 0.78 was confirmed in an adequately powered future trial, it would represent a clinically meaningful and statistically demonstrable reduction in mortality. TSA therefore supports the conclusion that a mortality benefit of MB in septic shock may exist but cannot yet be verified with the available evidence: the current body of RCTs is insufficient in size, not negative in direction.

Our findings should therefore be interpreted as reflecting uncertainty rather than futility. The available data support the concept that MB may be useful as an adjunctive hemodynamic therapy, particularly in vasoplegic shock with high vasopressor requirements [9,13,19], but they do not justify routine early use for outcome improvement. Future trials should be multicenter, adequately powered based on prospective sample size calculations, and designed to clarify the optimal timing, dose, duration, and target population for MB administration. Phenotypic enrichment strategies, for example, restricting enrollment to patients with confirmed nitric oxide-mediated refractory vasoplegia, may increase the probability of detecting a treatment effect. Consistent reporting of patient-centered outcomes, adverse events, and mechanistic biomarkers will be essential to advance the field.

## 5. Conclusions

In adults with circulatory shock, current randomized evidence does not demonstrate a statistically significant benefit of methylene blue on short-term mortality, renal replacement therapy, or length of stay. Although MB may improve hemodynamic parameters and reduce vasopressor exposure, the certainty of evidence remains insufficient to support routine clinical use. Trial sequential analysis demonstrated that only a fraction of the required information size of 1773 patients has been enrolled to date, and the cumulative Z-curve did not cross benefit, significance, or futility boundaries. The pooled OR of 0.73 (95% CI 0.40–1.36) reflects a consistent directional signal favoring MB that remains statistically inconclusive due to insufficient sample size, not due to evidence of absence of effect. Multicenter, adequately powered trials targeting the RIS are required before MB can be recommended as a routine adjunctive therapy in septic or vasoplegic shock.

## Figures and Tables

**Table 1:** Characteristics of included randomized controlled trials.

Author-Year	Country	Study Design	Population/Shock Type	N	Intervention (Dosage)	Control	Primary Outcome	Key Results	Follow-Up
**SEPTIC SHOCK**									
Kirov et al. (2001) [21]	Norway	RCT—Pilot, Open-label	Septic shock (Suspicion of infection, SIRS and end-organ dysfunction)	20 (10 MB/10 C)	MB 2 mg/kg bolus (15 min) + infusion 0.25, 0.5, 1 up to 2 mg/kg/h × 4 h each (total 5.75 mg/kg)	ISEV + ST (Vasopressor: dopamine or NE + fluid resuscitation guided with PAC + Dobutamine for CI > 3.5)	Hemodynamics & organ function at 24 h	↓ Vasopressor support ↑ O_2_ delivery No adverse effects Non-significant mortality difference	24 h
Memis et al. (2002) [16]	Turkey	RCT	Severe sepsis (Confirmed infection and ACCP/SCCM 1992 criteria)	30 (15 MB/15 C)	MB 3 mg/kg over 6 h (0.5 mg/kg/h × 6 h)	ISEV + fluid resuscitation guided by CVP (No other vasopressors or inotropes)	Cytokine levels at baseline, 24 h and 48 h	No difference in cytokines Transient ↑ MAP No survival benefit	48 h
Aguilar et al. (2016) [17]	Mexico	RCT	Septic shock (Suspicion of infection, SIRS and vasopressor requirement)	60 (30 MB/30 C)	MB 2 mg/kg bolus over 1 h	ISEV + ST (NE as needed + hydrocortisone 200 mg/24 h)	Hemodynamics, lactate, vasopressor dose, mortality	↑ Faster MAP improvement ↓ Lactate ↓ Vasopressor support ↓ Mortality	72 h
Ibarra-Estrada et al. (2023) [19]	Mexico	Single-center RCT	Septic shock (Sepsis-3 criteria, NE within 24 h)	91 (45 MB/46 C)	MB 100 mg/6 h daily × 3 days (~1.2 mg/kg/day)	ISEV + ST (US-guided fluid resuscitation + NE ± vasopressin if NE > 0.25 µg/kg/min + hydrocortisone 200 mg/day)	Time to vasopressor discontinuation at 28 days	↓ Time to vasopressor d/c (69 h vs. 94 h, *p* < 0.001) ↑ Vasopressor-free days ↓ ICU & hospital LOS No difference in mortality	96 h
Luis-Silva et al. (2024) [23]	Brazil	Pilot RCT (1:1 allocation)	Septic shock (Infection + MAP < 65 mmHg + lactate > 2 mmol/L + NE > 0.2 µg/kg/min)	42 (19 MB/23 C)	MB 3 mg/kg loading + 0.5 mg/kg/h × 48 h	ST (NE + vasopressin + hydrocortisone 200 mg/d + fluids + antibiotics)	Hemodynamics and vasopressor dose reduction	↓ Vasopressor dose Safe: no adverse events Cytokine levels NSD	72 h
Shaker et al. (2025) [18]	Egypt	RCT—Blinded	Septic shock (Sepsis-3 criteria; cancer patients)	90 (30 MB low/30 MB high/30 C)	High dose: 4 mg/kg bolus Low dose: 1 mg/kg bolus Both + 0.25 mg/kg/h × 72 h	ISEV + ST (Fluid resuscitation by ICOM + NE + hydrocortisone if NE > 0.2 µg/kg/min)	Hemodynamic response by dose	↓ Vasopressor dose and faster discontinuation ↓ Mortality (dose-dependent response) NSD in organ dysfunction Safety data reported	72 h
Dong et al. (2025) [24]	China	Single-center RCT (1:1)	Septic shock (Sepsis-3 criteria + mechanical ventilation)	72 (36 MB/36 C)	MB 2 mg/kg bolus (15 min) + 1 mg/kg infusion × 12 h	ISEV + ST	Sublingual microcirculation parameters at 24 h	↑ Microcirculatory parameters (HVM) No improvement in organ function or survival	24 h
Kuri et al. (2025) [20]	India	RCT	Septic shock (Sepsis-3 criteria, NE ≥ 0.2 µg/kg/min)	74 (37 MB/37 VA)	MB 1 mg/kg bolus (30 min) + 0.5 mg/kg/h × 6 h	Vasopressin 0.04 units/min × 6 h	NE dose at 6 h, 12 h & 24 h	NSD at 0–6 h VA group: ↓ NE at 12–24 h ↓ Lactate & SOFA at 24 h	24 h
**VASOPLEGIC/POST-CARDIAC SURGERY SHOCK**									
Levin et al. (2004) [22]	Argentina	RCT	Vasoplegic syndrome post-cardiac surgery (CPB): MAP < 50 mmHg, CVP < 5 mmHg, WCP < 10 mmHg, CI ≥ 2.5 L/min/m^2^, SVR < 800 dyn·s·cm^−5^, vasopressor use	56 (28 MB/28 C)	MB 1.5 mg/kg IV (single dose)	Placebo	Resolution of vasoplegic syndrome	Mortality: 0% MB vs. 21.4% placebo vs. resolved < 2 h No rebound vasoplegia	96 h

Abbreviations: C, control; CI, cardiac index; CPB, cardiopulmonary bypass; CVP, central venous pressure; HVM, heterogeneity of microvascular flow; ICOM, integrated cardiac output monitoring; ISEV, individualized supportive and evidence-based vasopressor; LOS, length of stay; MAP, mean arterial pressure; MB, methylene blue; NE, norepinephrine; NSD, no significant difference; PAC, pulmonary artery catheter; RCT, randomized controlled trial; SIRS, systemic inflammatory response syndrome; SOFA, Sequential Organ Failure Assessment; ST, standard treatment; SVR, systemic vascular resistance; US, ultrasound; VA, vasopressin; VS, vasoplegic syndrome; WCP, wedge capillary pressure.

**Table 2:** GRADE Summary of Findings: Methylene Blue versus Standard Therapy in Adult Circulatory Shock.

Outcome (No. of Studies; Participants)	Risk of Bias	Inconsistency	Indirectness	Imprecision	Certainty (GRADE)	Effect Estimate (95% CI)
28–30-day all-cause mortality (8 studies; *n* = 479)	Serious ^a^	Not serious (I^2^ = low)	Serious ^b^	Serious ^c^ (CI crosses null; n << RIS 1773)	⊕○○○ Very Low	OR 0.73 (95% CI 0.40–1.36)
Renal replacement therapy (2 studies; *n* = 131)	Serious ^a^	Not assessed (2 studies)	Serious ^b^	Very serious ^cd^ (2 studies only; very wide CI)	⊕○○○ Very Low	OR not estimable with precision
Hospital length of stay (3 studies; *n* = 222)	Serious ^a^	Serious ^b^ (clinical heterogeneity)	Serious ^b^	Serious ^c^ (wide CI; few studies)	⊕○○○ Very Low	SMD not statistically significant
ICU length of stay (3 studies; *n* = 222)	Serious ^a^	Serious ^b^ (clinical heterogeneity)	Serious ^b^	Serious ^c^ (wide CI; few studies)	⊕○○○ Very Low	SMD not statistically significant

^a^ Risk of bias: 4 studies at low risk, 2 with some concerns, 2 at high risk (RoB 2 tool); downgraded one level. ^b^ Indirectness/Inconsistency: heterogeneous shock phenotypes (septic vs. vasoplegic), variable MB dosing regimens, and different comparators; downgraded one level. ^c^ Imprecision: confidence intervals cross the null; accrued information size far below the required information size (RIS = 1773 patients per TSA); downgraded one level. ^d^ Additional downgrade for very serious imprecision (only 2 studies for RRT). CI, confidence interval; ICU, intensive care unit; LOS, length of stay; MB, methylene blue; OR, odds ratio; RIS, required information size; RRT, renal replacement therapy; SMD, standardized mean difference; TSA, trial sequential analysis. ⊕○○○, very low certainty of evidence (GRADE).

## Data Availability

The data analyzed in this study were extracted from published articles cited in the reference list. Additional extraction tables and analytic code should be made available by the corresponding author upon reasonable request.

## Supplementary Materials

The following supporting information can be downloaded at: https://www.mdpi.com/article/10.3390/jcm15124481/s1, Table S1. Detailed search strategies for each electronic database; Table S2. Full text review of excluded articles and reasons for exclusion [25,26,27,28,29,30,31,32].

## Abbreviations

The following abbreviations are used in this manuscript:

CI	confidence interval
cGMP	cyclic guanosine monophosphate
ICU	intensive care unit
MB	methylene blue
OR	odds ratio
PRISMA	Preferred Reporting Items for Systematic Reviews and Meta-Analyses
RCT	randomized controlled trial
RRT	renal replacement therapy
RoB 2	revised Cochrane risk-of-bias tool for randomized trials
SMD	standardized mean difference
TSA	trial sequential analysis
