# Supplementary material for: The Effectiveness of Methylene Blue in Adult Shock: A Systematic Review, Meta-Analysis, and Trial Sequential Analysis of Randomized Controlled Trials
Source: J Clin Med. 2026 Jun 10;15(12):4481. doi: 10.3390/jcm15124481 (PMC13302755; doi:10.3390/jcm15124481)
Supplement: Supplementary file 1 [file jcm-15-04481-s001.zip › jcm-4216869-supplementary.pdf]

Supplementary material

Table S1. Detailed search strategies for each electronic database

Data base	Research strategy	Date	Results
Medline #1	("Methylene Blue"[Mesh]<br>OR "methylene blue"[tiab]<br>OR "methylthioninium chloride"[tiab])<br>AND<br>("Shock"[Mesh]<br>OR shock[tiab]<br>OR "circulatory shock"[tiab]<br>OR "septic shock"[tiab]<br>OR "cardiogenic shock"[tiab]<br>OR "vasodilatory shock"[tiab]<br>OR "vasoplegic shock"[tiab]<br>OR vasoplegia[tiab]<br>OR "hypovolemic shock"[tiab]<br>OR "distributive shock"[tiab]<br>OR "obstructive shock"[tiab]<br>OR "anaphylactic shock"[tiab]<br>OR "neurogenic shock"[tiab]<br>OR "refractory shock"[tiab]<br>OR "postoperative shock"[tiab])	01/02/26	515
Medline #2	((((((Methylene blue[Title/Abstract]) AND (shock[Title/Abstract])) OR (septic shock[Title/Abstract])) OR (vasoplegia syndrome[Title/Abstract])) NOT (children)) NOT (neonates) AND (2007:2026[pdat])) NOT (case report)	01/02/26	189
Embase	#20 #3 AND #19 912<br>#19 #4 OR #5 OR #6 OR #7 OR #8 OR #9 OR #10 OR #11 OR #12 OR #13 OR #14 OR #15 OR #16 OR #17 OR #18 330450<br>#18 'postoperative shock':ab,ti,kw 178<br>#17 'refractory shock':ab,ti,kw 1561<br>#16 'neurogenic shock':ab,ti,kw 281<br>#15 'anaphylactic shock':ab,ti,kw 5925<br>#14 'obstructive shock':ab,ti,kw 612<br>#13 'distributive shock':ab,ti,kw 780<br>#12 'hypovolemic shock':ab,ti,kw 4741<br>#11 'vasoplegia' 1918<br>#10 'vasoplegia':ab,ti,kw 1239<br>#9 'vasoplegic shock':ab,ti,kw 356<br>#8 'vasodilatory shock':ab,ti,kw 693<br>#7 'cardiogenic shock':ab,ti,kw 37161<br>#6 'septic shock':ab,ti,kw 56313	03/02/26	912

	#5 'circulatory shock':ab,ti,kw 2008<br>#4 'shock':ab,ti,kw 329321<br>#3 #1 OR #2 30701<br>#2 'methylthioninium chloride':ab,ti,kw 119<br>#1 'methylene blue':ab,ti,kw 30633		
Cochrane Central	("methylene blue" OR methylthioninium OR methylthioninium chloride OR methylen* blue*)<br>AND<br>(shock* OR septic shock* OR septic* OR distributive shock* OR vasodilatory shock* OR cardiogenic shock* OR hypovolemic shock* OR hemorrhagic shock* OR anaphylactic shock* OR anaphylax* OR neurogenic shock* OR obstructive shock*)	01/02/26	88

Table S2. Full text review excluded articles and reasons for exclusion.

Reference	Reasons for exclusion
Juffermans et al. Nitric Oxide 22 (2010) 275–280(1)	Wrong study design
Mehaffey et al. Ann Thorac Surg 2017;104:36–41(2)	Wrong study design
Donati et al. Crit Care Med 2002 Vol. 30, No. 10 (3)	Wrong study design
Hiruy et al. J of Cardiothor and Vasc Anesth 37 (2023) 2228 2235(4)	Wrong study design
Gachot et al. Intensive Care Med (1995) 21:1027-1031(5)	Wrong study design
Yune, H et al. 1453: Crit Care Med 44(12):p 439, 2016.(6)	Wrong publication type
Delgadillo NA. Crit Care Med. 2023;51(1):22-22. (7)	Wrong publication type
Ponnappan KT, et al. InTransplantation 2018 May 1 (Vol. 102, pp. 38-39). (8)	Wrong publication type (No results published)

[1] Juffermans NP, Vervloet MG, Daemen-Gubbels CRG, Binnekade JM, Jong MD, Groeneveld ABJ. A dose-finding study of methylene blue to inhibit nitric oxide actions in the hemodynamics of human septic shock. Nitric Oxide. mayo de 2010;22(4):275-80. doi:10.1016/j.niox.2010.01.006

2. Mehaffey JH, Johnston LE, Hawkins RB, Charles EJ, Yarboro L, Kern JA, et al. Methylene Blue for Vasoplegic Syndrome After Cardiac Operation: Early Administration Improves Survival. Ann Thorac Surg. julio de 2017;104(1):36-41. doi:10.1016/j.athoracsur.2017.02.057
3. Donati A, Conti G, Loggi S, Münch C, Coltrinari R, Pelaia P, et al. Does methylene blue administration to septic shock patients affect vascular permeability and blood volume?: Crit Care Med. octubre de 2002;30(10):2271-7. doi:10.1097/00003246-200210000-00015
4. Hiruy A, Ciapala S, Donaldson C, Wang L, Hohlfelder B. Hydroxocobalamin Versus Methylene Blue for the Treatment of Vasoplegic Shock Associated With Cardiopulmonary Bypass. J Cardiothorac Vasc Anesth. noviembre de 2023;37(11):2228-35. doi:10.1053/j.jvca.2023.07.015
5. Gachot B, Bédos JP, Veber B, Wolff M, Régnier B. Short-term effects of methylene blue on hemodynamics and gas exchange in humans with septic shock. Intensive Care Med. diciembre de 1995;21(12):1027-31. doi:10.1007/BF01700666
6. Yune H, Kim K, Jo YH, Kim J, Lee JH, Chung H, et al. 1453: INFUSION OF METHYLENE BLUE IN SEVERE SEPSIS AND SEPTIC SHOCK: A RANDOMIZED CONTROLLED TRIAL. Crit Care Med. diciembre de 2016;44(12):439-439. doi:10.1097/01.ccm.0000510127.34774.13
7. Delgadillo NA, Ibarra-Estrada M, Sandoval-Plascencia L, López-Pulgarin JA, Chavez-Peña Q, Mijangos-Mendez J, et al. 44: ADJUNCTIVE METHYLENE BLUE FOR PATIENTS IN SEPTIC SHOCK: THE SHOCKEM-BLUE TRIAL. Crit Care Med. enero de 2023;51(1):22-22. doi:10.1097/01.ccm.0000906052.35064.ea
8. KT Ponnappan, Pandey CK, Maiwall R, Saluja V, Tandon M, Parvez MQ, et al. Evaluating in cirrhotics with refractory vasoplegia the effect of methylene blue (crumbs)-a randomized controlled trial. InTransplantation. mayo de 2018;102:38-9.
